# Ochratoxigenic Black Species of Aspergilli in Grape Fruits of Northern Italy Identified by an Improved PCR-RFLP Procedure 

**DOI:** 10.3390/toxins4020042

**Published:** 2012-01-30

**Authors:** Davide Spadaro, Subban Patharajan, Alessia Lorè, Angelo Garibaldi, Maria Lodovica Gullino

**Affiliations:** 1 Centre of Competence for the Innovation in the Agro-environmental Sector, Università degli Studi di Torino, Via L. da Vinci 44, Grugliasco (TO) I-10095, Italy; Email: subban.patharajan@unito.it (S.P.); alessia.lore@unito.it (A.L.); angelo.garibaldi@unito.it (A.G.); marialodovica.gullino@unito.it (M.L.G.); 2 DiVaPRA-Plant Pathology, Università degli Studi di Torino, via L. da Vinci 44, Grugliasco (TO) I-10095, Italy

**Keywords:** *Aspergillus *section *Nigri*, grapes, ochratoxin A, ribosomal DNA, wine

## Abstract

A collection of 356 isolates of *Aspergillus* spp. collected during 2006 and 2007 from grapevines in northern Italy were identified through Internal Transcribed Spacer based Restriction Fragment Length Polymorphism (ITS-RFLP) and tested for ochratoxin A (OTA) production. Restriction endonuclease digestion of the ITS products using the endonucleases *Hha*I, *Hinf*I and *Rsa*I, distinguished five different RFLPs. From each pattern, three samples were sequenced and the nucleotide sequences showed different species corresponding to *Aspergillus niger*, *A. carbonarius*, *A. tubingensis*, *A. japonicus* and *A. aculeatus*. By comparing the sequences of the ITS regions, also the uniseriate species *A. japonicus* and *A. aculeatus* could be differentiated by *Hinf*I digestion of the ITS products. Among the aspergilli, *A. niger* was the major species associated with grapes during 2006 (57.4%), while *A. carbonarius* was the major species during 2007 (46.6%). All the strains of *Aspergillus *were tested for their ability to produce OTA on Yeast extract sucrose medium (YES), as it was tested as an optimal substrate for the evaluation of OTA production by black aspergilli. Out of 356 isolates, 63 (17.7%) isolates produced OTA ranging from 0.05 to 3.0 µg mL^−1^. Most of the ochratoxigenic isolates were *A. carbonarius *(46) in both years, but also some strains of *A. tubingensis* (11) and *A. japonicus* (6) produced lower amounts of OTA.

## 1. Introduction

Ochratoxin A (OTA) is a major mycotoxin in grapes and grape products [1,2,3]. OTA is nephrotoxic, hepatotoxic, genotoxic, teratogenic and immunotoxic to animals and its carcinogenicity in rats and mice is well-established [4]. It has been associated with the aetiology of Balkan Endemic Nephropathy [5] and the development of tumours in the urinary tract in humans [6]. The International Agency for Research on Cancer classified OTA as a possible carcinogen to humans (group 2B) [7]. The contamination of grapes and their by-products by OTA has emerged as a big problem for the health risk related to the consumption of such products by human beings [8,9]. The European Union has introduced limits (2 μg L^−1^) for OTA in wine and grape products in order to minimize exposure to OTA in the diet (EC Regulation No 1881/2006). Ochratoxin A is produced during the infection on grapes in vineyards by toxigenic species of black aspergilli belonging to *Aspergillus* section *Nigri*, in particular *A*. *niger* and *A*. *carbonarius* [10,11,12]. *A. carbonarius* is predominantly responsible for the production of OTA in grapes and wine [2,13,14,15], followed by some isolates of the *A. niger* aggregate [16]. In the present study, *Aspergillus niger *and *Aspergillus tubingensis* were considered two separate species according to Kusters-van Someren *et al*. [17]. Some authors claim the production of OTA by *A. japonicus *[18], however such ability is still unconfirmed. The percentage of *A. niger* aggregate isolates able to produce OTA is much lower than the share of *A. carbonarius* isolates [19]. The main OTA contamination in grapes and wine was reported in different countries but high levels of OTA were found in southern Europe [18,20], in particular in regions with a Mediterranean climate [21,22,23,24].

An accurate identification of the species of *Aspergillus* is of great importance because the toxin profiles of each species vary, and the fungi present limit and define potential toxicological risks. Black aspergilli are difficult to classify and the taxonomy of this section is still unclear [25,26]. Various molecular approaches have been proposed, though the taxonomy of *Aspergillus* section *Nigri* is not completely resolved, especially within the *A. niger* aggregate [26,27,28,29]. Among the molecular tools proposed, restriction fragment length polymorphism (RFLP) has been used successfully to identify *Aspergillus* species [26]. Those findings were confirmed by RFLP on genomic DNA with different endonucleases, combined with rDNA hybridization probes [30], by RFLP on mtDNA [31,32], and by RAPD analysis [30]. Accensi *et al*. [33] proposed a new PCR-RFLP method to distinguish the *A. niger* aggregate and identified two types, N (corresponding to *A. niger*) and T (corresponding to *A. tubingensis*). More recently, a new group of ochratoxigenic strains, closely related to *A. tubingensis*, has been identified by ITS-RFLP method [34]. Moreover, some strains belonging to the uniseriate species *Aspergillus aculeatus* and *A*. *japonicus* showed to produce OTA [21], but their PCR-RFLP profile was not able to differentiate the two species.

The first objective was to monitor the population of black aspergilli in Northern Italian vineyards during the year 2006 and 2007, and the second objective was to assess the mycotoxigenicity of all the isolates. The collateral objective of this study was the optimization of a restriction fragment length polymorphism (RFLP) method to distinguish the five species of *Aspergillus* present on grapes cultivated in Northern Italy. 

## 2. Materials and Methods

### 2.1. Collection of Grape Samples and Isolation of *Aspergillus* spp.

During 2006 and 2007, grape samples were collected at harvesting stage from Albenga, Liguria region, Italy. The isolates were collected from grape bunches belonging to six different grapevine varieties (Lumassina, Rossese, Vermentino, Ormeasco, Pigato and Vermentino 84). At harvest, 10 bunches were picked from 10 different plants located approximately along two diagonals crossing the vineyard. Each bunch was collected in a separate paper bag and samples were brought to the laboratory. Five berries were taken randomly from each bunch and they were surface decontaminated using a 0.1% sodium hypochlorite solution for 30 s followed by two rinses with sterile-distilled water. The samples were plated on Potato dextrose agar (PDA, Merck) dishes containing streptomycin 50 mg L^−1^ (Merck). Plates were incubated at 25 °C for 7 days. The fungal colonies were picked up and transferred to PDA slants for further studies. The initial identification of isolates of *Aspergillus *spp. was achieved through macroscopic and microscopic observation with the aid of guidelines [35]. Other genera of fungi, isolated during the experiments, were not considered in the present study. Some reference strains of *Aspergillus* spp. were obtained from the Centralbureau voor Schimmelcultures (CBS, Utrecht, The Netherlands): CBS 113.80 (*Aspergillus carbonarius*); CBS 107.55 (*Aspergillus tubingensis*); CBS 113.46 (*Aspergillus niger*); CBS 116.80 (*Aspergillus aculeatus*); and CBS 114.51 (*Aspergillus japonicus*).

### 2.2. Culture Conditions and Extraction of DNA

For the extraction of DNA, all the strains of *Aspergillus *spp. were grown in Yeast extract sucrose (YES) broth at 25 °C for 9 days. Then, the mycelia were collected through Whatman No.1 filter paper. Three hundred mg of mycelium from the individual strains was frozen in liquid nitrogen and ground to a fine powder. DNA extractions were performed with 100 mg of powder using the commercial NucleoSpin Plant DNA kit (Macherey-Nagel, Iuren, Germany) according to the manufacturer’s instructions. 

### 2.3. PCR Conditions and DNA Digestions

The 5.8 S-ITS region was amplified by PCR using universal primers ITS 5 and ITS 4 [36]. PCR reactions were performed in 20 μL as the final volume, containing 3 μL of 10× buffer (with 2.5 mmol L^−1^ MgCl_2_), 1 μL of 2 mmol L^−1^ dNTP mixture, 3 μL of 2 mol L^−1^ each primer, 0.2 μL (1 U) of Taq DNA polymerase (Invitrogen, USA), 7.8 μL of dH_2_O and 2 μL of (50 ng) DNA template. The reaction mixtures were performed in a Thermocycler (Biometra, Germany) with 35 cycles consisting of 1 min at 95 °C, 1 min at 52 °C and 2 min at 72 °C and the final extension time with 10 min. Then, the PCR products were purified by using Qiagen (Valencia, CA, USA) PCR purification kit and digested with the restriction enzymes *Hha*I, *Hinf*I and *Rsa*I (Promega, Madison, WI, USA). Each 20 μL reaction mixture contained 2 μL of 10X reaction buffer, 0.2 μL of BSA (10 μg μL^−1^), 0.5 μL of restriction enzyme (10 U μL^−1^), 5 μL of purified DNA and 12.3 μL of dH_2_O and incubated at 37 °C for 2 h. The restriction fragments were separated on 2% agarose gel at 50 V, stained SYBR-safe (Invitrogen) and photographed using a Gel Doc 1000 system. Size of the restriction fragments were estimated by comparison with a marker (50 bp DNA ladder-Gelpilot, Qiagen).

### 2.4. Sequencing Analysis

The PCR products of three samples for each RFLP pattern were sequenced. They were purified using a QIAquick PCR purification kit (Qiagen) and measured with a spectrophotometer. They were cloned with a pDrive cloning vector using the Qiagen cloning kit according to manufacturer’s instructions. Plasmids were purified from bacterial cells following manufac–turer’s instruction (Qiagen) and were sequenced by the BMR Genomics Centre (Padova, Italy) using the ABI PRISM 3730Xl DNA Sequencer. The ITS sequences from the different *Aspergillus* spp. were submitted to the GenBank of the National Centre for Biotechnology Information (NCBI, New York, NY, USA) with the following accessions: GQ118984, GQ359404, GQ359405 (*A*. *carbonarius*); GQ118985, GQ359406, GQ359407 (*A*. *niger*); GQ129209, GQ359408, GQ359409 (*A*. *tubingensis*), GQ129210, GQ359410, GQ359411 (*A*. *aculeatus*); GQ129211, GQ359412, GQ359413 (*A*. *japonicus*). The 5.8S-ITS region sequences were aligned using the multiple sequence alignment program CLUSTAL X. The genetic distances were calculated using the Jukes-Cantor model and the phylogenetic inference was obtained by UPGMA (Unweighted Pair Group Method with Arithmetic Mean) method. The statistical confidence of a particular group of sequences in the tree, evaluated by bootstrap test (10,000 pseudo replicates) [38], using the computer program MEGA version 2.0 [39]. 

### 2.5. Production of OTA by *Aspergillus* spp.

The isolates of *Aspergillus* spp.—all of them were black aspergilli–were tested for their ability to produce OTA on flasks containing 300 mL YES broth. For the production of OTA the isolates were grown for 9 days by shaking (100 rpm) at 25 °C. Three ml of the culture filtrates were withdrawn and centrifuged at 10,000 rpm for 10 min. Then, each sample was diluted ten times with distilled water and OTA was extracted from the individual culture filtrate using Ochra Test^TM^ columns (Vicam, Watertown, USA) as per the manufacturer instructions. Samples were analyzed in a HPLC Agilent series 1100 formed by a degasser, an autosampler, a quaternary pump, a thermostated column and a fluorimeter. An analytical column RP-18 (150 mm × 4.6 mm i.d., 5 μm) with a pre-column was used. The mobile phase, eluting at 1 mL min^−1^, consisted of an isocratic mixture of acetonitrile:water:acetic acid (45:45:10) for 18 min. 100 μL of sample were injected onto the HPLC column and the retention time of OTA was 6.15 min. The amount of OTA in the final solution was determined by using a calibration graph of concentration versus peak area and expressed as ng/mL, achieved by injection onto the HPLC column of 100 μL of standard solutions of OTA (Sigma Chemical Co.). The standard solutions had concentrations of 0.5, 1.0, 5.0, 10.0, 25.0, 50.0 and 100.0 ng mL^−1^. The recovery was determined on a blank YES broth spiked at three concentrations of OTA (0.1, 2.0 and 10.0 ng mL^−1^). Samples were processed for estimation of OTA as described above. Recovery experiments were done in four replicates. Recoveries ranged from 90.8% to 92.1%. The repeatability ranged from 2.64% to 2.71% for replicate analysis. The detection limit of the analysis was 0.01 ng OTA mL^−1^ of YES medium. 

## 3. Results

### 3.1. ITS-RFLP of *Aspergillus* Isolates

**Figure 1:**
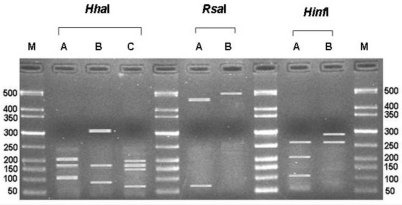
Ribosomal DNA restriction patterns exhibited by *Aspergillus* isolates from grapes after digestion with the restriction endonucleases *Hha*I, *Rsa*I and *Hinf*I. Lane M corresponds to the 50 bp molecular weight marker (Gelpilot, Qiagen).

During a two year survey, we could isolate from the grapes coming from Liguria region (Italy) and identify 356 isolates of *Aspergillus* spp. based on morphological characteristics. The other genera of fungi isolated were not considered in the present study. All the isolates were characterized at species level using an ITS-RFLP based method. The size of all amplified PCR products was estimated at around 546–596 bp. On the basis of the ITS sequences, the endonucleases *Hha*I, *Hinf*I and *Rsa*I were chosen for the restriction analysis in order to differentiate among isolates. Their typical restriction patterns are shown in Figure 1. 

The individual profiles designated with the letters A–C, can be combined into composite restriction patterns or RFLP types (Table 1). The restriction patterns obtained for the different isolates were compared with those obtained from the reference strains of *Aspergillus* species. The analysis of the *Aspergillus* isolates showed five different RFLP types (Table 1).

**Table 1:** Ribosomal restriction patterns and composite patterns exhibited by the *Aspergillus* isolates analyzed in the present study.

Type	Species	No of isolates	Restriction patterns & size of the fragments (bp)		
			*Hha*I	*Rsa*I	*HinfI*
N	*A. niger*	153	A	A	A
			207, 178, 114	480, 66	269, 202, 110
C	*A. carbonarius*	107	B	A	B
			318, 178, 91	480, 66	294, 270
T	*A. tubingensis *	72	A	B	A
			207, 178, 114	500	269, 202, 110
A	*A. japonicus *	20	C	A	B
			185, 174, 156, 75	480, 66	294, 270
J	*A. aculeatus*	4	C	A	A
			185, 174, 156, 75	480, 66	269, 202, 110

**Figure 2:**
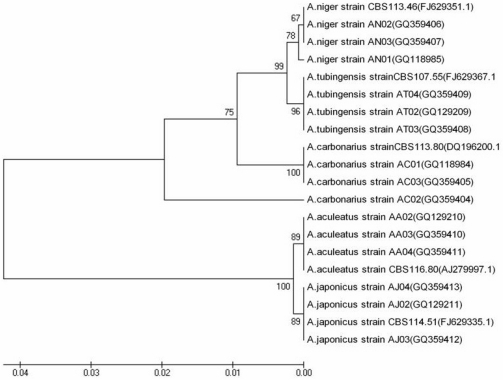
Phylogenetic tree constructed for 15 isolates of *Aspergillus* spp. and 5 Centralbureau voor Schimmelcultures (CBS) reference strains of *Aspergillus* spp. by the Unweighted Pair Group Method with Arithmetic Mean (UPGMA) Methods from 5.8S-ITS sequences, aligned using the multiple sequence alignment program CLUSTAL X. The genetic distances were calculated using the Jukes-Cantor model. Numbers at the nodes represent the proportion of 10,000 bootstrap samples, in which a clade was found.

The phylogenetic relationship between the isolates of *Aspergillus* species is illustrated in a UPGMA cluster analysis (Figure 2). Three sequences from each *Aspergillus* species were perfectly aligned with the CBS reference strains. The ITS sequences strongly identified two main groups with 100% bootstrap value: uniseriate black aspergilli were clearly separated from the biseriate black aspergilli. The isolates of *A. tubingensis* and *A. niger* were separated into two subdivisions (99% bootstrap value) and clustered with the CBS reference strains. Within the isolates *A. carbonarius*, the strain AC02 showed a slightly different sequence but it clustered with the other isolates of *A. carbonarius*. The sequence of AC02, when blasted in NCBI, showed 100% homology with *A. carbonarius *and just 91% homology with *A. ibericus*. More interestingly, *A. japonicus* and *A. aculeatus* were further divided into two subgroups (89% bootstrap value) and aligned perfectly with the CBS reference strains.

### 3.2. OTA Producing Ability of *Aspergillus* Isolates

All the 356 isolates of *Aspergillus* spp., identified through RFLP, were tested for their ability to produce OTA in YES medium. HPLC analysis showed that 63 isolates were able to produce OTA (17.7% of the total population: Table 2). Among these 63 isolates, 46 belonged to the species *A. carbonarius* and produced high amount of OTA ranging from 0.3–3.0 μg mL^−1^. Eleven isolates of *A. tubingensis* produced OTA ranging from 0.05-0.8 μg mL^−1^, and 6 isolates of *A. aculeatus* produced 0.05–0.4 μg mL^−1^ of OTA. The isolates of *A. niger* and *A. japonicus* were not able to produce OTA. 

**Table 2:** Ochratoxin A (OTA) production by *Aspergillus *spp. isolated from grapes during 2006–2007.

Species	Total No. of isolates and percentage		No of OTA producers (%)		OTA content (range µg mL^−1^)
	2006	2007	2006	2007	
*A. niger*	(57.4%) 112	(25.5%) 41	0	0	ND
*A. carbonarius*	(16.4%) 32	(46.6%) 75	11 (34.4%)	35 (46.7%)	0.30 ± 0.10–3.0 ± 0.60
*A. tubingensis*	(14.4%) 28	(27.3%) 44	6 (21.4%)	5 (11.4%)	0.05 ± 0.01–0.8 ± 0.38
*A. japonicus*	(9.7%) 19	(6.2%) 1	5 (26.3%)	1 (100.0%)	0.05 ± 0.02–0.4 ± 0.09
*A. aculeatus*	(2.1%) 4	(0.0%) 0	0	0	ND
Total	195	161	22 (11.3%)	41 (25.5%)	-

ND, Not detected; ±, standard deviation.

## 4. Discussion

In the present study restriction digestion analysis of the ITS products was tested to assess its effectiveness as a rapid method to identify different species of *Aspergillus* isolated from grapes comparing with reference strains. The other fungal genera isolated were not considered in the present study. The ITS-5.8S rDNA fragments of 356 *Aspergillus* strains isolated from grapes were amplified and their PCR amplicons were digested in order to differentiate the strains using the RFLP technique. Five different RFLP patterns were identified among the *Aspergillus* isolates using the endonucleases *Hha*I, *Hinf*I and *Rsa*I. By comparing the sequence analysis of the 356 isolates with that of some reference strains, all the isolates showed high similarity (>99%) with one of the five reference species and were considered as isolates of the species *A. niger*, *A. carbonarius*, *A. tubingensis*, *A. japonicus*, or *A. aculeatus*. No other species of *Aspergillus *were identified during the monitoring. 

In a previous study, Martínez-Culebras and Ramón [34] digested the ITS region of several strains of *Aspergillus *coming from Spanish grapes with the endonucleases *Hha*I, *Nla*III, and *Rsa*I, and distinguished five different RFLP patterns corresponding to *A. niger*, *A. tubingensis*, *A. tubingensis*-like, *A. carbonarius*, and *A. aculeatus*. They obtained two restriction profiles using endonuclease *Rsa*I. One profile was specific for *A*. *niger*, *A*. *aculeatus *and *A*. *carbonarius*, and the second profile identified *A*. *tubingensis*. Similar results were obtained in the present study by using the endonuclease *Rsa*I. Similarly, Accensi *et al*. [33] described the restriction endonuclease *Rsa*I as a useful tool to separate the isolates of *A. niger* aggregate into *A. niger* (corresponding to pattern N) and *A. tubingensis* (corresponding to pattern T). The results of the current study confirmed that the endonuclease *Rsa*I can be used as a target for distinguishing the isolates of *A. niger* and *A. tubingensis*. This classification supports previous RFLP studies suggesting the division of *A. niger* aggregate into over two species, including *A. niger* and *A. tubingensis* [33,34,40].

The endonuclease *Hha*I distinguished three profiles [34]: profile A (specific for *A*. *niger*, *A*. *tubingensis*-like and *A*. *tubingensis*), profile B (*A*. *carbonarius*), and profile C (*A*. *aculateus*). The isolates *A*. *tubingensis*-like and *A*. *tubingensis* were not distinguished with the endonuclease *Rsa*I and *Hha*I, and an additional endonuclease *Nla*III was proposed for the new RFLP type that was called *A. tubingensis*-like. In the present study we could not differentiate the species *A*. *aculeatus* and *A*. *japonicus* with endonuclease *Rsa*I and *Hha*I. An alternative restriction enzyme *Hinf*I was used successfully to distinguish the two uniseriate species. Previously, Parenicová *et al*. [41] reported that the ITS-5.8S ribosomal region could not be used to distinguish between *A. japonicus* and *A. aculeatus*. Anyway, the same authors, by using Southern blotting with hybridization probes on the pyruvate kinase (*pkiA*), the pectine lyase A (*peIA*) and the 28S rRNA, found a clear polymorphism between *A. japonicus* and *A. aculeatus* [41]. Similarly, Palencia *et al.* [42] reported the potential of a barcoding tool based on the rep-PCR, to differentiate between the two *Aspergillus* species. Our PCR-RFLP method can simply and easily differentiate five species of *Aspergillus*, including *A. japonicus* and *A. aculeatus*, present on grapes. 

The phylogenetic analysis based on the 5.8S rRNA gene and the non-coding and variable regions (ITS1 and ITS2) showed that the isolates of five different *Aspergillus* species coming from Liguria region clustered with the CBS reference strains belonging to the same species. The isolates of *A. tubingensis* and *A. niger* were clearly separated into two distinct groups within the *A. niger* aggregate. These results are consistent with the work of Martínez-Culebras *et al*. [41], who recently used the ap-PCR method to distinguish *A. tubingensis* and *A. niger*, and with previous studies [14,33,34,40]. More interestingly, the ITS sequences of the two uniseriate species of *A. aculeatus* and *A. japonicus* within the *Aspergillus* section *Nigri* were separated into two different subgroups by cluster analysis. Martínez-Culebras *et al*. [41] reported that the isolates of *A. aculeatus* and *A. japonicus* could not be separated by ITS or IGS sequences. 

In our 2-year survey, we monitored the presence of isolates of *Aspergillus *spp. associated with vineyards grown in Northern Italy. The absolute dominance of black aspergilli on grapes before harvesting was reported by other surveys in other countries, including Italy [21,23,43], France [44], Argentina [45] and Spain [34,46]. No other species of *Aspergillus *were isolated and identified in our study. Among the black aspergilli, *A. niger* was the major species associated with grapes during 2006, while *A. carbonarius* was the major species during 2007. All the cultivars monitored possessed the same species and the same order of dominance over the two year period. We considered the average meteorological data of Liguria region (Italy) during the 2006 and 2007 trimesters characterized by grapevine ripening and harvest (July, August and September). The average temperatures in July, August and September 2006 were 26.0, 21.5 and 20.2 °C, while the same in 2007 were 22.7, 21.3 and 19.1 °C. By considering the rainfall of the trimester, in 2006 the total precipitation reached 546 mm, while it accounted for just 182 mm in 2007. The first year was characterized by higher temperatures on an average, especially in July, and three times more precipitation. These climatic conditions could have influenced the composition of the black aspergilla found in vineyard. Maximum growth for *A. carbonarius* occurred at 30 °C, while it occurred at higher temperatures (35 °C) for *A. niger* [26,47]. Also the water activity (a_w_) could influence the maximum growth that occurred at *a*_w_ values of 0.965 *a*_w_ for *A. carbonarius* and 0.98 for *A. niger* [48].

YES medium was chosen as it was previously evaluated as an optimal substrate for the evaluation of OTA production on black aspergilli [49]. Other studies considered the production of OTA by species of *Aspergillus *on other substrates, such as CYA (Czapek Yeast Extract agar). By a comparison of different *Aspergillus *isolates grown on YES or CYA, both culture media could be used for evaluating the OTA production [50].

Most of the ochratoxigenic strains were belonging to *A. carbonarius* in both years. This result confirmed previous surveys carried out in Italy from 2000 to 2003 showing that *A. carbonarius* was never the dominant species in any phonological phase of grapevine, but its isolates were the most toxigenic ones in different grape varieties grown in several Italian regions [1,14]. Similar results were obtained in other European countries where a lower share of *A. carbonarius* was responsible for most of the OTA contamination [22,34,51]. Ochratoxin A was measured during 2006 and 2007 on the wine produced from the grapes monitored, and the level of contamination was generally low, but higher during 2007 (0.15 µg L^−1^) compared to 2006 (0.09 µg L^−1^). Within the *Aspergillus niger *aggregate, no ochratoxigenic isolates of *A. niger* were detected, but 6 and 5 isolates of *A. tubingensis*, respectively in 2006 and 2007, were able to produce low amounts of OTA *in vitro*. The percentages of isolates of *A. tubingensis *able to produce OTA were 21.4% and 11.4% in 2006 and 2007. Our results are in accordance with Medina *et al*. [52] and Martínez-Culebras and Ramón [34], but another study of Accensi *et al*. [53] showed that the isolates producing OTA belonged to *A. niger*, while none of the isolates of *A. tubingensis* was able to produce OTA. Recently some studies showed that also isolates of *A. tubingensis* can produce ochratoxin A [27,41]. The geographical origin of the isolates might be a reason for the disagreement in the results reported by different authors. Experimental conditions used in extraction and detection of OTA (culture medium, pH, incubation time and/or temperature) might also lead to different result. The ability of uniseriate black aspergilli to produce OTA is a controversial issue. In our study, 6 isolates of *A. japonicus *out of 20 were ochratoxigenic, and our result is similar to the results of Battilani *et al. *[21].

The availability of reliable molecular markers for *Aspergillus* section *Nigri* occurring on grapes is of great interest since an early diagnosis of *A. carbonarius *occurrence in the field would provide very important information on possible OTA contamination in grapes. In conclusion, the identification of a large number of isolates using ITS-RFLP technique enables easy and rapid identification of the *Aspergillus* isolates from grapes and their assignation to different species. This kind of information could help to understand the epidemiology and distribution of OTA producing *Aspergilli* in grapes, where large amounts of isolates should be screened in a short time.
